# Telemedicine Support for Primary Care Providers versus Usual Care in Patients with Heart Failure: Protocol of a Pragmatic Cluster Randomised Trial within the Brazilian Heart Insufficiency with Telemedicine (BRAHIT) Study

**DOI:** 10.3390/ijerph20115933

**Published:** 2023-05-24

**Authors:** Leonardo Graever, Aurora Felice Castro Issa, Viviane Belidio Pinheiro da Fonseca, Marcelo Machado Melo, Gabriel Pesce de Castro da Silva, Isabel Cristina Pacheco da Nóbrega, Leonardo Cançado Monteiro Savassi, Mariana Borges Dias, Maria Kátia Gomes, Jose Roberto Lapa e Silva, Raphael Mendonça Guimarães, Renato Cony Seródio, Anne Frølich, Henrik Gudbergsen, Janus Christian Jakobsen, Helena Dominguez

**Affiliations:** 1 Departamento de Clínica Médica, Faculdade de Medicina, Universidade Federal do Rio de Janeiro, Rio de Janeiro 21044-020, Brazil; 2 Department of Biomedical Sciences, Faculty of Health and Medical Sciences, University of Copenhagen, 2200 Copenhagen, Denmark; lamora@sund.ku.dk; 3 Instituto Nacional de Cardiologia, Rio de Janeiro 22240-006, Brazil; auroraissa@gmail.com (A.F.C.I.); viviane.belidio@gmail.com (V.B.P.d.F.); mmmelo@icloud.com (M.M.M.); redcap.inc.rj@gmail.com (I.C.P.d.N.); 4 Departamento de Medicina de Família, Saúde Mental e Coletiva, Universidade Federal de Ouro Preto, Minas Gerais 35400-000, Brazil; leosavassi@gmail.com; 5 Ministry of Health, Brasilia 70058-900, Brazil; mariana.borges@saude.gov.br; 6 Escola Nacional de Saúde Pública, Rio de Janeiro 21041-210, Brazil; raphael.guimaraes@fiocruz.br; 7 Secretaria Municipal de Saúde, Rio de Janeiro 20211-110, Brazil; 8 Department of Public Health, Faculty of Health and Medical Sciences, University of Copenhagen, 1353 Copenhagen, Denmark; anfro@sund.ku.dk (A.F.); henrik.gudbergsen@sund.ku.dk (H.G.); 9 Copenhagen Trial Unit, Centre for Clinical Intervention Research, Capital Region of Denmark & Department of Regional Health Research, The Faculty of Health Sciences, University of Southern Denmark, 2200 Copenhagen, Denmark; janus.jakobsen@ctu.dk

**Keywords:** primary health care, telemedicine, heart failure, clinical competence, cluster randomized trial

## Abstract

Heart failure is a prevalent condition and a frequent cause of hospital readmissions and poor quality of life. Teleconsultation support from cardiologists to primary care physicians managing patients with heart failure may improve care, but the effect on patient-relevant outcomes is unclear. We aim to evaluate whether collaboration through a novel teleconsultation platform in the Brazilian Heart Insufficiency with Telemedicine (BRAHIT) project, tested on a previous feasibility study, can improve patient-relevant outcomes. We will conduct a parallel-group, two-arm, cluster-randomised superiority trial with a 1:1 allocation ratio, with primary care practices from Rio de Janeiro as clusters. Physicians from the intervention group practices will receive teleconsultation support from a cardiologist to assist patients discharged from hospitals after admission for heart failure. In contrast, physicians from the control group practices will perform usual care. We will include 10 patients per each of the 80 enrolled practices (n = 800). The primary outcome will be a composite of mortality and hospital admissions after six months. Secondary outcomes will be adverse events, symptoms frequency, quality of life, and primary care physicians’ compliance with treatment guidelines. We hypothesise that teleconsulting support will improve patient outcomes.

## 1. Introduction

Heart failure is a common outcome of many cardiovascular and metabolic diseases, leading to a high disease burden, frequent hospital admissions, and poor quality of life [1]. The adequate management of patients with heart failure relies on care integration among healthcare sectors [2,3,4], including primary care, especially in low- and middle-income countries, where it is known to reduce admission rates [5,6,7]. One recommended collaboration strategy is teleconsultation (e-consultation) support between healthcare providers, usually from subspecialists to primary care physicians [8,9,10].

Previous observational studies have shown that teleconsultation support enhances primary care physicians’ confidence, capacity, and satisfaction rates [8,11]. In cardiology, Olayiwola et al. [12] suggested that teleconsultation may increase access to cardiology evaluation while reducing in-person referrals. On the other hand, two extensive reviews [13,14] showed that no study clearly demonstrated the impact of provider-to-provider communication using technology on patient-relevant outcomes such as mortality, hospital admissions, or quality of life.

Although the use of remote communication tools between providers is already recommended by the World Health Organisation [10], one must ascertain whether this technology is effective and impacts patient-relevant outcomes to guide rational stakeholders’ investment in teleconsultation services.

In this study, we aim to test the effects of a teleconsultation service provided by the BRAHIT project, a Danish-Brazilian academic collaboration, on heart failure management in primary care practices in Rio de Janeiro, Brazil. We will perform a cluster randomised trial to account for the practice-level intervention effect. The primary outcome will be a composite of hospital readmission and mortality. Secondary outcomes will be serious adverse events, patient-reported quality of life, and frequency of symptoms. The physicians’ compliance with treatment guidelines will be reported as an exploratory outcome.

## 2. Materials and Methods

### 2.1. Study Design

We will conduct a parallel-group, two-arm, superiority cluster-randomised controlled trial with a 1:1 allocation ratio. 

### 2.2. Study Setting

The study setting is the primary healthcare network of Rio de Janeiro, the second-largest city in Brazil, with 6,320,446 inhabitants and a free public health system [SUS]. There are 236 primary care practices with 1307 health teams composed of one physician, one nurse, one nursing technician, and four to six community health agents delivering comprehensive care for inhabitants of a defined area, according to the family health strategy model [15,16,17]. Besides this conformation, there are also practices where primary care is not entirely delivered following this model, but by physicians and allied professionals who are not integrated into the team. 

The population can access the health system at their corresponding practice, pre-hospital facilities (*Unidade de Pronto Atendimento*—UPA), or the emergency rooms of secondary hospitals. When a patient in primary care needs further evaluation in an acute setting, the patient is transported by ambulance to a pre-hospital facility or an emergency room in a secondary hospital, where they can remain in observation or be admitted to the ward or intensive care unit. Tertiary hospitals, reserved for high-complexity cases, are accessed through regulation systems managed by the state administration.

Data on heart failure epidemiology and management in Rio de Janeiro is scarce, usually found only in surveys and academic publications. If we estimate a 1% prevalence in the general population (63,204 people living with heart failure), an admission rate of 24.9% and a mortality rate of 24% per year in this specific population, as found in the literature [18,19,20], one could expect around 15,378 admissions and 3777 deaths per year in the city. 

We started the Danish-Brazilian BRAHIT project in 2019 using telemedicine tools to improve primary care in heart failure. In a single-arm feasibility test, we assigned cardiologists to support primary care physicians from one practice on heart failure case management via videoconference meetings at physicians’ requests. Twenty-four cases were discussed through videoconference. This model was used until June 2021, when it was scaled up and adapted to a web-based platform, where 40 new patients were included from 11 other primary care practices and specialised hospital-based homecare teams. Data from the 64 cases revealed some improvement opportunities. According to the physicians’ reports provided with the teleconsultation requests, most patients were in poor physical status, with 51% experiencing NYHA (New York Heart Association) class III or IV symptoms. In addition, 67% had at least 2 signs or symptoms of poor clinical control, i.e., dyspnoea, dizziness, fatigue, sleeping problems, leg swelling, pulmonary rales, or jugular distension. Essential recommended therapy was prescribed for only 19% of patients, and only 14% had their vital signs adjusted within the recommended limits (blood pressure < 120 × 80 mmHg and heart rate < 80 bpm).

### 2.3. Eligibility

#### 2.3.1. Inclusion Criteria for Patients

Patients over 18 years old with a diagnosis of heart failure who have been discharged within the past month from a public hospital or pre-hospital facility and are registered in a public primary care practice.

#### 2.3.2. Inclusion Criteria for Study Centres and Staff

All 236 primary care practices from Rio de Janeiro municipality and their physicians are eligible for the study. According to the above criteria, we will include primary care practices progressively, determined by where the eligible patients are registered. 

#### 2.3.3. Exclusion Criteria

We will exclude participants who refuse to participate in the study, patients where heart failure is excluded as a diagnosis after the first contact with primary care or teleconsultation, and patients without a defined reference physician to receive the teleconsultation intervention, due to physician shortage. We will also exclude from the study the practices and patients involved in the feasibility test.

### 2.4. Interventions

The intervention comprises teleconsultation support from a cardiologist to a primary care physician in reference to the case management of a patient included in the study.

Based on the primary discharge diagnosis, the research team will collect admissions due to decompensated heart failure, from monthly reports provided by the municipality. We will invite the eligible patients and their corresponding primary care physicians to participate in the study. Invitations will be issued by texting, telephone call, or in-person contact, and signed informed consent forms from patients and physicians will be obtained. 

After the patient and physician agree to participate, we will sign up the physician on a secure online platform for teleconsultation hosted on the research team’s web domain. Through a digital form, they will provide data about professional identification, contact information, patient demographics, and clinical data. They will use the practice’s computers or their own devices to connect and discuss the case with the teleconsulting cardiologist.

The consultant cardiologists must be active National Institute of Cardiology staff members and perform academic activities in undergraduate or graduate programs. They are trained in heart failure management and in using the project’s digital tools. They will use the computers from the research department at the National Institute of Cardiology (INC) or personal devices to access the platform at least twice a week on working days and give feedback to primary care physicians about clinical management. 

The interactions may happen asynchronously, via the teleconsultation platform’s message interface or WhatsApp© texting, or synchronously, by phone call or videoconference, at the physicians’ preference. The duration of the teleconsultation period will depend on the physician’s judgment. This approach is designed to ensure the pragmatism of the intervention [21,22]. Besides the changes recommended by the teleconsultation, all patients will receive the usual care planned by their practice teams, including physician and nurse consultations, prevention measures, oral health treatment, and community agent follow-up visits.

### 2.5. Participant Timeline and Study Workflow

The schedule of enrolment, interventions, and assessments is systematised in Table 1. 

**Figure 1:**
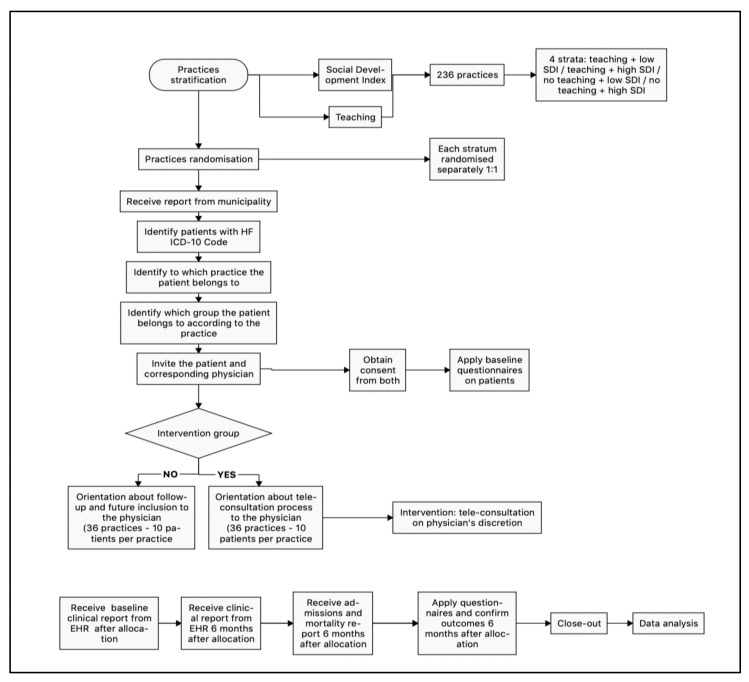
Study workflow.

The study workflow, including data collection and management, is illustrated in Figure 1.

### 2.6. Recruitment (Patient and Provider)

A recruitment researcher (recruiter) will receive once monthly a database of admissions from the municipality Health Secretariat, including patients with a registered heart failure ICD-10 code (I11.0, I13.0, I42.0, I42. 6, I42.9, I50. 0, I50.1 or I50.9) who have been discharged during the previous month. Within one week, they will identify the primary care practice where the patient is registered, according to the patient’s address, in the public database *“Onde Ser Atendido”* [23] and invite the correspondent physician, who is responsible for the care of the patient, to participate in the study, by e-mail, telephone, or in person at the practice. When the physician accepts, the recruiter invites the patient by telephone or in person. We expect to invite all discharged patients from the municipality database (an average of 127 patients per month) and a 10% refusal rate, with an estimated recruitment rate of 114 patients/month, lasting for eight months, from March to September 2023. The recruitment will stop once the target sample size of 80 clusters with 10 patients each is reached.

The recruiter will obtain consent forms from patients and physicians, deliver orientation about the study process, and monitor the recruitment rate weekly. No financial incentive will be provided for the enrolment either of patients or physicians.

### 2.7. Assignment of Interventions

#### 2.7.1. Allocation—Sequence Generation

Primary care practices from Rio will be stratified into four strata according to two factors, each having two levels: the family health strategy model running exclusively in the practice (presence or absence) and the Social Development Index (≤0.60 or >0.60). [24]. The research team has concluded that both factors are important determinants of the work process quality and patient outcomes, respectively [5,25,26].

We will randomise practices within each stratum after recruitment to control or intervention groups, using a computerised random number generator on a simple 1:1 allocation ratio to increase homogeneity between the groups. Once we randomise the practices, we will allocate the patients and apply the intervention of the physician or follow-up, depending on the group.

#### 2.7.2. Allocation—Concealment Mechanism 

The practices and patient allocation will be revealed only to researchers directly recruiting and assigning interventions (e.g., teleconsultation). The allocation will be concealed from the rest of the research team.

#### 2.7.3. Allocation—Implementation

An independent collaborator who does not participate in conducting the trial will receive the list of the practices to be stratified and randomised. After randomisation, they will send the recruiter an updated list containing allocation information. Based on the monthly report received from the Health Secretariat, the recruiter will allocate the patients in groups according to their registered practice. The researchers who are responsible for allocating and assigning interventions will not be involved in the intervention. Once one cluster reaches ten patients, the assignment for that cluster stops, in order to ensure equal cluster sizes.

### 2.8. Blinding

Blinding of care providers is not possible, because those in the intervention group will receive teleconsultation support. The patients, outcome assessors, recruiters, data managers, and the data safety monitoring committee will be sought blinded. 

Two independent blinded statisticians will perform statistical analyses with the two intervention groups coded ‘A’ and ‘B’. The steering group will draw two blinded conclusions: one assuming ‘A’ is the experimental group and ‘B’ is the control group—and one assuming the opposite. Based on these two blinded conclusions, two abstracts will be written (and published as a supplement to the primary publication). When the blinding is broken, the ‘correct’ abstract will be chosen, and the conclusions in this abstract will not be revised.

### 2.9. Data Retention

Once a patient and the corresponding physician and practice are recruited, the research team will apply and monitor the retention rate. We will disseminate the project in social media to increase awareness, adhesion, and retention of the primary care physicians in the project. 

In the intervention group, the teleconsulting team will promptly respond to physicians’ requests, use communication tools that best suit their routine, and consider the practice and patient’s context to deliver a feasible teleconsultation. Those features were assessed during the feasibility test, with favourable results. These factors will increase participant and data retention. A dedicated researcher will monitor the teleconsulting process, ensuring that physicians evenly receive high-quality support. Discontinuation of participants’ adherence after enrolment will be used for censoring and analysed following an intention-to-treat principle.

### 2.10. Data Management

The research team has a secure REDCap© environment hosted at the National Institute of Cardiology of Brazil server, where databases and forms will be created and hosted, comprising data from primary care practices and physicians collected from public websites, randomisation lists, reports shared by the municipality about hospital admissions, primary care clinical data, mortality data, recruitment assignments and monitoring, and the answers to patient-reported outcomes questionnaires. A database and a reporting system have been developed within the e-consultation platform for management of intervention data, hosted at a secure web domain contracted by the project team. 

Researchers will only have access permission to data concerning their specific activities (e.g., recruiter, teleconsultant, data analyst) on REDCap© and the e-consultation platforms. Access will be granted through individual passwords changed regularly. The data manager will perform a weekly scheduled backup to a different server. 

For analysis, all data will be merged into one single database on the study’s REDCap© environment, based on the patients’ single identifier numbers (CPF). The data management team will monitor missing data and apply measures to communicate and minimise any lack. We will store the complete database for one year after the end of the study and share anonymised datasets in a dataset repository. 

### 2.11. Statistical Analysis

#### 2.11.1. Sample Size

We calculated the sample size based on the primary outcome, considering the expected values and differences between groups as described in an extensive European survey of heart failure morbidity, which reports a composite index of readmissions and mortality of 40.1% per year [19]. The estimated effect size based on similar interventions described in the literature is 30% [27]. Assuming an intra-cluster correlation (ICC) of 0.05 based on similar studies in primary care [28], a minimum of 72 clusters of 10 patients in each cluster are necessary to detect a 15% difference in the primary outcome with 95% significance (5% type I error) and 80% power (20% type II error). Based on this sample size, we have estimated the power of all remaining outcomes.

For calculations, we used the sample size calculator for cluster randomized trials by the Health Services Research Unit from Aberdeen University [29] and the R software package “CRTsize”.

#### 2.11.2. General Analysis

We will conduct all analyses according to the intention-to-treat principle, where we analyse data as randomised regardless of adherence to the research protocol. An additional per-protocol analysis will be performed for exploratory reasons if the drop-out rate exceeds 5% of the total trial population. We will assess whether the statistical and clinical significance thresholds apply [30], based on the estimated intervention effects and power estimations used in the sample size.

We will base our main conclusions on the primary outcome, following the intention-to-treat principle, assuming a type 1 error threshold of 5% (*p* < 0.05), with the relative risk and confidence intervals (CIs) calculated at the 95% level and a type 2 error threshold of 0.2 (80% statistical power).

Regression analyses will be adjusted for the factors used in the randomisation (the exclusive presence of the family health strategy model in the practice and the social development index stratum), assessing for significant interactions between them and the outcome variables. We will consider the results of secondary or exploratory outcomes as hypothesis-generating only. No subgroup analysis besides the one using the stratification factors is planned; if executed, these results will also be hypothesis-generating only.

Categorical variables will be described as absolute and relative frequencies, and continuous variables as medians (interquartile range) or means (standard deviation) according to the distribution of the variable. Missing data will be handled according to Jakobsen’s recommendations [31]; briefly, we will include participants in the primary analysis of all outcomes and anticipate a less than 5% missing value rate. We will then perform a secondary analysis considering the use of multiple imputations and present best-worst and worst-best case scenarios if it is not valid to ignore missing data.

To account for clustering within each primary care practice, all outcomes will be analysed using generalised estimating equation methods (GEE) [32]. All analyses will additionally be adjusted for the stratification variables used in the randomisation. The software R (R Foundation for Statistical Computing, Vienna, Austria; version x64 3.6.0, including the package “geepack” for GEE) [33] and STATA will be used for statistical analysis.

### 2.12. Study Duration

Based on the hospitals’ discharge frequency, we estimate recruiting patients for six months and collecting data for another six months after reaching the target sample and cluster size; therefore, the study will last one year.

### 2.13. Ethics and Dissemination

The BRAHIT study protocol is approved by Brazil’s National Ethics Committee, with the number 14894819.5.0000.5272. All patient data will be followed up by the research team throughout the study, in addition to usual care, upon municipality authorisation. There will be no interference in the cases’ management apart from the designed actions of the study. During recruitment, we will request consent via a written consent form (Annex) from all physicians and patients involved in the clinical trial. The project is registered in the domain www.clinicaltrials.gov under the number NCT04466852.

## 3. Results

### 3.1. Data Collection Methods

We will collect primary care practices’ data to assess stratification factors (presence of family health strategy exclusively in the practice and population’s socioeconomic status) from Rio’s publicly accessible health system websites [16,34,35,36]. We will obtain a monthly report from the Health Secretariat about hospital admissions containing name, birth date, social identification number (CPF), national health system identification number (CNS), address, hospital, admission date, admission diagnosis, outcome (discharge/transference/death), discharge diagnosis, discharge date, and procedures. Data about the patient’s primary care practice (name, address, phone number, responsible physician) will be collected from the public website *Onde Ser Atendido* [23].

The recruiters will collect baseline data about the physicians through direct inquiry during recruitment. For patient data, we will receive a report extracted from primary care electronic health records including patients’ clinical data, completed during patient recruitment through direct inquiry, to minimise missing data. We will insert data using a REDCap © platform digital form. Baseline collected variables and a description of form fields are summarised in Table 2.

In the intervention group, during teleconsultation, the primary care physician and the teleconsultant (cardiologist) will insert data about the process using a specific form on a digital e-consultation platform designed for the project. The form contains the same fields as the REDCap© form, the reason for teleconsultation, and boxes for text messages. Data from the e-consultation platform will be exported to REDCap© for storage and analysis. In the control group, the recruiter will inform the physician that data from patients with heart failure who were recently discharged from the practice will be collected at baseline and after six months of allocation. We will train all the physicians in using the e-consultation platform.

During recruitment, interviewers assigned to the project will apply paper questionnaires about quality of life (EQ-5D-5L questionnaire) [37,38,39] and heart failure symptoms frequency (Symptom Status Questionnaire—SSQ-HF) [40,41] for baseline assessment.

After six months of the allocation, we will repeat the described data collection for admissions, serious adverse events, and primary care electronic health records and re-apply EQ-5D-5L and SSQ-HF questionnaires. We will collect mortality data from a report on mortality provided by the Health Secretariat.

### 3.2. Outcomes

The primary outcome will be a composite of all-cause mortality or one or more hospital readmissions. Any pre-hospital or hospital stay because of heart failure will be considered as a readmission [42,43]. Secondary outcomes will include hospital-free days within 180 days [44,45,46], serious adverse events (defined as any adverse event that results in death, is life-threatening, requires hospitalisation or prolongation of existing hospitalisation, or results in persistent or significant disability or incapacity, measured in municipality admission reports), frequency and intensity of heart failure signs and symptoms, measured using the Symptom Status Questionnaire—Heart Failure—Brazilian Version (Appendix A) [40,41], computed as a 0 to 84 score, and health-related quality of life, measured using the EQ-5D-5L questionnaire [37,38] scored using a value set validated for the Portuguese version [39] on a visual-analogic scale (0 to 100).

An exploratory outcome will be physicians’ adherence to guidelines, comparing prescription data to guidelines and quality standards using a compliance score (Table A1 in Appendix B) [47,48,49,50]. We will measure all outcomes at the patient level, at baseline when meaningful, and after six months of allocation.

## 4. Risks and Harms

The main risk of the project is related to the use rate of the teleconsulting system by primary care professionals, which might be lower than expected. However, the experience gathered during the BRAHIT feasibility test based on over 60 patients managed using the teleconsultation approach described above indicates that the project is feasible in the planned period for its execution.

Other risks include changing the BRAHIT project team at the institutional level; this risk is deemed low, as they are professionals admitted through public examination in their home institutions. The interaction of graduate students with pre-planned teaching programs will reduce the project risk. The study intervention has a low yield of inflicting harm on participants. Teleconsultations may prevent patients from being seen by a specialist; this has been described as a risk in systematic reviews [51], although the issue has not yet been addressed in studies to date.

## 5. Discussion

The study addresses a knowledge gap relating to the effectiveness and safety of teleconsultation support for primary care physicians. The study topic is its application to support the clinical management in primary care services of patients with uncompensated heart failure who were recently discharged from hospitals in Rio de Janeiro, Brazil.

E-health tools, including telemedicine, teleconsultation, and telemonitoring, have been used increasingly, especially since the COVID-19 pandemic. Aspects such as ethical and medical-legal issues of e-health interventions are currently under study and debate [52], so it is justified that we carefully assess the effectiveness of these interventions to allow a proper evaluation of risk-to-benefit ratio.

We expect to analyse the effects of the teleconsultation support from cardiologists to primary care physicians on patient-relevant outcomes, such as all-cause mortality, hospital readmission, patient-reported quality of life, symptom frequency, serious adverse events, and compliance with guidelines by the primary care physicians. The study will address these multiple outcomes using a pragmatic approach recommended for the study of public health interventions [53,54,55].

Many studies highlight difficulties concerning diagnosis and compliance with heart failure treatment guidelines in primary care settings [56,57,58,59,60]. We believe these hurdles may be due to primary care physicians’ competence gap in managing heart failure patients.

Our experience from a still unpublished feasibility test indicated improvement opportunities, reinforcing the need described in the reviewed literature [13,14] for a clinical trial with a rigorous methodology to assess teleconsultation effectiveness using patient-relevant outcomes.

Some factors limit our study. Although designed as a randomised clinical trial, many pragmatic features place it at an intermediate point within the PRECIS-2 explanatory–pragmatic continuum [22]. We anticipate some heterogeneity in the cluster population. Our eligibility criteria are broad and include all primary care practices from Rio de Janeiro, regardless of the primary care model (family health strategy versus non-family strategy organisations); we used stratification techniques to minimise this effect. We will include all adult patients with confirmed heart failure of all aetiologies, regardless of comorbidities or clinical status. We will analyse data according to the intention-to-treat principle, despite the primary care physicians’ adherence to the intervention. Although these factors aim to increase our findings’ external validity, they may undermine statistical analysis. We will rely on stratification and randomisation to overcome bias and anticipate data loss through recruitment and retention monitoring.

Notwithstanding its limitations, our research may produce valid and reliable data to guide recommendations for public policy implementation that may lead to primary care quality improvement in heart failure management and possibly other chronic conditions, in line with recommendations from national and global institutions [10,61] which advocate the use of e-Health tools to improve primary care quality.

## 6. Conclusions

Teleconsultation between specialists and primary care physicians may have a potential role in improvement of heart failure management in primary care. If proven effective, the collaboration model can be further studied and utilised for other conditions, especially in locations with scarce professional human resources.

## Figures and Tables

**Table 1:** Participant Timeline.

Enrolment	T_−1_	T_0_	T_6_ (Close-Out)
General practice stratification and randomisation	X		
Patient eligibility screening using municipality data	X		
Invitation to participate (patient)	X		
Invitation to participate (physician)	X		
Informed consent (patient and physician)	X		
Patient allocation according to practice randomisation		X	
Intervention			
Intervention group		Teleconsultation support for the practice’s physicians (first contact and on demand)	
Control group		Usual care with follow-up of outcomes	
Assessments		T_0_	T_6_ (close-out)
1—Readmissions and Mortality (reports)			X
2—Days Alive Out of Hospital (DAOH)			X
3—Frequency of Serious Adverse Events			X
4—Symptoms Frequency and Severity		X	X
5—Quality of Life		X	X
6—Adherence to Prescribing Guidelines		X	X

**Table 2:** Baseline Variables.

N	Variable	Field Type	Choices, Calculations, or Labels
Physician Variables—direct inquiry			
1	Name	Text	-
2	Social Identification Number (CPF)	Text	-
2	Completed years since graduation	Number	-
3	Specialisation	Option	Any/Family Medicine/Other
4	Completed years in primary care	Number	-
5	Completed years in the current practice	Number	-
6	Enrolled in a residency program	Options	No/Preceptor/1st year Resident/2nd year Resident/3rd year Resident/Program
Patient Variables—primary care electronic health record report			
1	Name	Text	-
2	Social identification number (CPF)	Text	-
3	National Health System identification number (CNS)	Text	-
4	Primary care practice	Text	
5	Gender	Options	Feminine/Masculine/Nonbinary
6	Race	Options	White/Black/Brown/Other
7	Birth date	Date	-
8	Weight (Kg)	Number	-
9	Height (cm)	Number	-
10	NYHA status	Options	1 to 4
11	Systolic blood pressure	Number	-
12	Diastolic blood pressure	Number	-
13	Heart rate	Number	-
14	Dyspnoea	Option	Yes/No
15	Leg Swelling	Option	Yes/No
16	Dizziness	Option	Yes/No
17	Fatigue	Option	Yes/No
18	Orthopnoea or paroxysmal nocturnal dyspnoea	Option	Yes/No
19	Syncope	Option	Yes/No
20	Typical chest pain	Option	Yes/No
21	Jugular vein distension	Option	Yes/No
22	Pulmonary rales	Option	Yes/No
23	ACE-I (names)	Option	ACE-I names
24	ACE-I dose	Number	-
25	ARB (names)	Option	ARB names
26	ARB dose	Number	-
27	Beta-blockers (names)	Option	BB Names
28	Beta-blockers dose	Number	-
29	Spironolactone	Option	Yes/No
30	Spironolactone dose	Number	-
31	Furosemide	Option	Yes/No
32	Furosemide dose	Number	-
33	Digoxin	Option	Yes/No
34	Digoxin dose	Number	-
35	Syncope	Option	Yes/No
36	Hypertension	Option	Yes/No
37	Diabetes	Option	Yes/No
38	Dyslipidaemia	Option	Yes/No
39	Coronary artery disease	Option	Yes/No
40	Peripheric artery disease	Option	Yes/No
41	Cerebrovascular disease	Option	Yes/No
42	Atrial fibrillation	Option	Yes/No
43	Chagas disease	Option	Yes/No
44	Pulmonary chronic obstructive disease	Option	Yes/No
45	Creatinine	Number	-
46	Echocardiogram ejection fraction	Number	-

ACE-I—angiotensin conversion enzyme inhibitors; ARB—angiotensin receptor blockers; BMI—body mass index; NYHA—New York Heart Association.

## Data Availability

The data presented in this study are available on request from the corresponding author. The data are not publicly available due to sensitive content.

## Appendix A. Symptom Status Questionnaire—Heart Failure

Instructions: Please read each statement carefully, then circle the number that best describes your condition or how much you were bothered by these symptoms during the past four weeks.

1. Did you have shortness of breath during the daytime?

0. No (If your answer is no—please go to question 2)

1. Yes (If your answer is yes—please fill out 1a, 1b, 1c)

  1a. How often?

  (1) Less than once per week   (2) 1–2 times per week   (3) 3–5 times per week   (4) Nearly daily

  1b. How severe?

  (1) Slight   (2) Moderate   (3) Severe   (4) Very severe

  1c. How much did it distress or bother you?

  (0) Not at all   (1) A little bit   (2) Somewhat   (3) Quite a bit   (4) Very much

2. Did you have shortness of breath when you lay down?

0. No (If your answer is no—please go to question 3)

1. Yes (If your answer is yes—please fill out 2a, 2b, 2c)

  2a. How often?

  (1) Less than once per week   (2) 1–2 times per week   (3) 3–5 times per week   (4) Nearly daily

  2b. How severe?

  (1) Slight   (2) Moderate   (3) Severe   (4) Very severe

  2c. How much did it distress or bother you?

  (0) Not at all   (1) A little bit   (2) Somewhat   (3) Quite a bit   (4) Very much

3. Did you have fatigue or lack of energy?

0. No (If your answer is no—please go to question 4)

1. Yes (If your answer is yes—please fill out 3a, 3b, 3c)

  3a. How often?

  (1) Less than once per week   (2) 1–2 times per week   (3) 3–5 times per week   (4) Nearly daily

  3b. How severe?

  (1) Slight   (2) Moderate   (3) Severe   (4) Very severe

  3c. How much did it distress or bother you?

  (0) Not at all   (1) A little bit   (2) Somewhat   (3) Quite a bit   (4) Very much

4. Did you have chest pain?

0. No (If your answer is no—please go to question 5)

1. Yes (If your answer is yes—please fill out 4a, 4b, 4c)

  4a. How often?

  (1) Less than once per week   (2) 1–2 times per week   (3) 3–5 times per week   (4) Nearly daily

  4b. How severe?

  (1) Slight   (2) Moderate   (3) Severe   (4) Very severe

  4c. How much did it distress or bother you?

  (0) Not at all   (1) A little bit   (2) Somewhat   (3) Quite a bit   (4) Very much

5. Did you have leg or ankle swelling?

0. No (If your answer is no—please go to question 6)

1. Yes (If your answer is yes—please fill out 5a, 5b, 5c)

  5a. How often?

  (1) Less than once per week   (2) 1–2 times per week   (3) 3–5 times per week   (4) Nearly daily

  5b. How severe?

  (1) Slight   (2) Moderate    (3) Severe    (4) Very severe

  5c. How much did it distress or bother you?

  (0) Not at all   (1) A little bit   (2) Somewhat   (3) Quite a bit   (4) Very much

6. Did you have difficulty sleeping at night?

0. No (If your answer is no—please go to question 7)

1. Yes (If your answer is yes—please fill out 6a, 6b, 6c)

  6a. How often?

  (1) Less than once per week    (2) 1–2 times per week   (3) 3–5 times per week   (4) Nearly daily

  6b. How severe?

  (1) Slight   (2) Moderate   (3) Severe   (4) Very severe

  6c. How much did it distress or bother you?

  (0) Not at all   (1) A little bit   (2) Somewhat   (3) Quite a bit   (4) Very much

7. Did you have dizziness or loss of balance?

0. No (If your answer is no—just check No and stop here)

1. Yes (If your answer is yes—please fill out 7a, 7b, 7c)

  7a. How often?

  (1) Less than once per week   (2) 1–2 times per week   (3) 3–5 times per week   (4) Nearly daily

  7b. How severe?

  (1) Slight   (2) Moderate   (3) Severe   (4) Very severe

  7c. How much did it distress or bother you?

  (0) Not at all   (1) A little bit   (2) Somewhat   (3) Quite a bit   (4) Very much

Scoring method and interpretation

1. Sum all scores of a, b, and c for each symptom. For example, dizziness score = 7a + 7b + 7c.

2. If a person did not experience the symptom, score 0 for all the symptom items.

3. Thus, the possible score range for each symptom will be 0 to 12. These seven combined scores will be used to obtain Cronbach’s alpha.

4. To obtain the total symptom score, sum all seven combined scores. Thus, the total possible score range is 0 to 84 (12*7).

5. Higher scores indicate more severe heart failure symptoms.

Copyright, 2014 Seongkum Heo, RN, PhD, Debra K. Moser, RN, PhD, and Terry Lennie, RN, PhD

## Appendix B. Treatment Adherence Score

**Table A1:** Treatment Adherence Score.

**Item/Points**	**2**	**1**	**0**
ACE-I use	Target Dose	Used below 50% of Target Dose [49]	Not used
OR			
ARB use	Target Dose	Used below 50% of the Target Dose	Not used
AND			
Beta-blockers use	Target Dose	Used below 50% of the Target Dose	Not used
Spironolactone use	Not indicated or Target Dose	Used below 50% of the Target Dose	Not used

Range: 0 to 8 points. ACE-I—Angiotensin-converting Enzyme Inhibitor; ARB—Angiotensin Receptor Blocker.
